# Co‐Cultivated Enzyme Constraint Metabolic Network Model for Rational Guidance in Constructing Synthetic Consortia to Achieve Optimal Pathway Allocation Prediction

**DOI:** 10.1002/advs.202306662

**Published:** 2023-12-13

**Authors:** Boyuan Xue, Yu Liu, Chen Yang, Hao Liu, Qianqian Yuan, Shaojie Wang, Haijia Su

**Affiliations:** ^1^ Beijing Key Laboratory of Bioprocess and Beijing Advanced Innovation Center for Soft Matter Science and Engineering Beijing University of Chemical Technology Beijing 100029 P. R. China; ^2^ Biodesign Center Key Laboratory of Engineering Biology for Low‐carbon Manufacturing Tianjin Institute of Industrial Biotechnology Chinese Academy of Sciences Tianjin 300308 P. R. China

**Keywords:** metabolic network models, pathway allocation, rational construction, synthetic consortia

## Abstract

Synthetic consortia have emerged as a promising biosynthetic platform that offers new opportunities for biosynthesis. Genome‐scale metabolic network models (GEMs) with complex constraints are extensively utilized to guide the synthesis in monocultures. However, few methods are currently available to guide the rational construction of synthetic consortia for predicting the optimal allocation strategy of synthetic pathways aimed at enhancing product synthesis. A standardized method to construct the co‐cultivated Enzyme Constraint metabolic network model (CulECpy) is proposed, which integrates enzyme constraints and modular interaction scale constraints based on the research concept of “independent + global”. This method is applied to construct several synthetic consortia models, which encompassed different target products, strains, synthetic pathways, and compositional structures. Analyzing the model, the optimal pathway allocation and initial inoculum ratio that enhance the synthesis of target products by synthetic consortia are predicted and verified. When comparing with the constructed co‐culture synthesis system, the normalized root mean square error of all optimal theoretical yield simulations is found to be less than or equal to 0.25. The analyses and verifications demonstrate that the method CulECpy can guide the rational construction of synthetic consortia systems to facilitate biochemical synthesis.

## Graphical abstract

The construction of biosynthetic pathways through synthetic consortia resembles a flexible combination of complex transportation modes. Development of a metabolic network simulation prediction method for guiding the rational allocation of complex pathways.

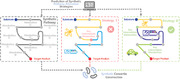

## 1 Introduction

With the rapid development of synthetic biology technology, biosynthesis has gradually become an important way for the green manufacturing of biochemicals. Researchers have successfully achieved the biosynthesis of most chemicals using inexpensive carbon sources through the combination of biological reactions and metabolic pathways.^[^ ^1^, ^2^ ^]^ Although monoculture systems are prevalent in biosynthesis, with the complexity of biosynthetic pathways, substrate composition, and the demand for high throughput of target products, monoculture systems can no longer fully meet the demand for efficient synthesis.^[^ ^3^, ^4^, ^5^ ^]^ Therefore, synthetic consortia (also referred to as co‐culture systems) have emerged as new platforms for biosynthesis of the target product,^[^ ^6^ ^]^ which allow more complex metabolic pathways and rationally assign them to different strains.

Synthetic consortia consist of two or more distinct strains and select appropriate intermediate metabolites as nodes to distribute complex metabolic pathways.^[^ ^7^, ^8^ ^]^ This approach aims to reduce the metabolic burden and adapt to complex substrate structures to improve the efficiency of target product synthesis. Synthetic consortia have been constructed and applied to the biosynthesis of various biochemicals, all with improved synthetic yields compared to monoculture systems.^[^ ^8^, ^9^, ^10^, ^11^, ^12^ ^]^ However, when employing synthetic consortia for biosynthesis, researchers must thoroughly investigate the impact of various complex conditions on the yield of the target product. This involves the exploration of how the intricate synthetic pathways within each module can be strategically allocated to achieve the highest possible synthetic efficiency in the co‐culture system, which cannot be achieved by existing modeling methods. It is evident that a greater number of new approaches are required to address these challenges, to predict the optimal pathway allocation strategies, and further enhance the efficiency of synthetic consortia construction.

In particular, the genome‐scale metabolic model (GEM) is proven to be a powerful computational tool to predict cellular metabolism and physiological states within living organisms,^[^ ^13^ ^]^ and the study of monoculture systems in combination with GEM for metabolic engineering is well‐established. By analyzing models, researchers can predict the yield of target metabolites and the flux of key reactions, optimize culture conditions, and successfully build efficient synthesis systems for important chemicals, such as lysine^[^ ^14^ ^]^ and succinic acid.^[^ ^15^ ^]^ Therefore, it is important to understand how to achieve dynamic control of complex compositional structures in synthetic consortia using complex constraints in metabolic network models^[^ ^16^, ^17^, ^18^ ^]^ to predict the optimal synthetic pathway allocation strategies for co‐culture systems.

However, genome‐scale metabolic models (GEMs) derived from synthetic consortia are not well‐rounded. Most published models only focus on interactions between colonies,^[^ ^19^, ^20^, ^21^ ^]^ or incorporate metabolomic data to study correlative interactions between strains.^[^ ^22^ ^]^ Although some models were capable of predicting flux distributions, they often neglect the primary objective of setting product synthesis and guiding the rational construction of synthetic consortia. Methods for these models have employed stoichiometric ratio constraints as crucial limitations to construct GEMs of microbial communities and predict microbial abundances, such as MICOM,^[^ ^23^ ^]^ OptCom,^[^ ^24^ ^]^ and SteadyCom.^[^ ^25^ ^]^ Currently, existing models developed for microbial communities rarely integrate enzyme constraint limitations, which hinders their abilities to accurately simulate the flux of key reactions and guide the rational construction of synthetic consortia.

**Figure 1:**
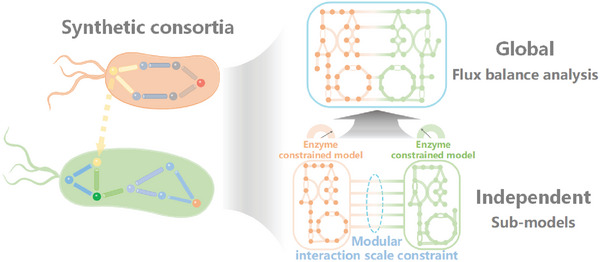
The “independent + global” synthetic consortia: Constructing complete sub‐models separately and integrating co‐cultivated enzyme constraints into each of these sub‐models. Global: Constructing co‐culture metabolic models and incorporating modular interaction scale constraints to perform global flux balance analyses

Therefore, we propose a novel “independent + global” concept to achieve the rapid construction of co‐cultivated enzyme constraint metabolic network (**Figure** **1**). The concept consists of two components. First, we constructed sub‐models for the synthetic consortia by creating comprehensive and independent metabolic model response networks for each strain. We incorporated co‐cultivated enzyme constraints to each sub‐model to ensure the independence of each sub‐model and enhance the accuracy of their simulations. This step is based on the “independent” concept of synthetic consortia model (SCM) construction. Then, we establish reactions connecting external metabolite fluxes between strains and incorporate new modular interaction scale constraints, integrating them into a large‐scale metabolic network model for global flux balance analyses. This step incorporates the “global” concept of analyzing SCM.

In this paper, a new method named CulECpy (co‐cultivated enzyme constraint metabolic network model based on python) was presented by adopting this “independent + global” concept for the rapid construction of enzyme‐constrained metabolic network models of synthetic consortia. We selected several sets of realistic synthetic consortia to verify the feasibility. The model integrated enzymatic constraints for co‐cultivated and modular interaction scale constraints, leading to a more realistic simulation of the multi‐strain synthetic pathway. This method has the capability to predict and assess the optimal pathway allocation strategy for the co‐culture synthesis system along with determining the most advantageous inoculation ratio. The CulECpy offers a powerful tool for guiding the rational construction of synthetic consortia for biochemicals.

## 2 Results

### 2.1 Construction of Synthetic Consortia Metabolic Network Model based on “Independent + Global” Research Idea

The design and construction of a synthetic consortium is different from the monoculture system. Researchers need to consider not only the genetic modification of individual strains, but also explore the selection of optimal strains, complex interactions between strains, and optimal allocation of synthetic pathways for synthetic consortia.^[^ ^8^, ^9^, ^10^, ^11^ ^]^ To meet these challenges, our construction methods need to be adapted to the desired chassis and meet the need for rapid modeling. We propose a novel method called CulECpy. This modeling method is based on the “independent + global” design concept. This method effectively solves key problems such as optimal pathway allocation strategies and strain inoculation ratios, and has excellent adaptability to complex synthetic consortia, which can significantly improve the efficiency of system construction.

#### 2.1.1 Setting the Interaction Reaction Between Sub‐Models

The model must have the ability to simulate the growth of strains in synthetic consortia and contain complete sub‐models with flux flow between them. Therefore, it is essential for SCM to consider interactions between strains and the actual growth state of each strain, which is the basis for model construction and simulation analysis.^[^ ^26^, ^27^ ^]^ To achieve this, new models can be constructed by splicing traditional GEMs.

**Figure 2:**
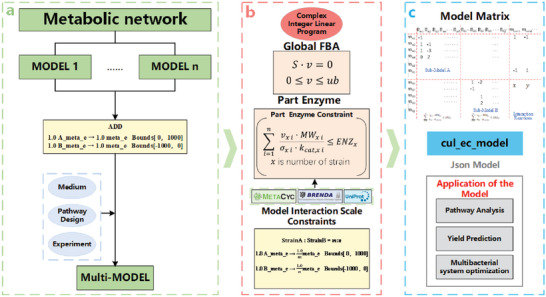
CulECpy workflow for construction of co‐cultivated enzyme constrained metabolic network model. a) Construct the synthetic consortia model (SCM) with stoichiometric constraints and set interaction reactions between sub‐models. b) Integrate multiple constraints for the SCM. c) Save synthetic consortia mode file and guide the rapid construction of synthetic consortia.

**Figure** **2** shows the workflow of CulECpy. In the first step, monoculture GEMs were used as the model materials, which are the published GEMs of different strains depending on the design of the synthetic consortia. In some co‐culture systems, the strains may be genetically modified and genetic information in the model files need to be corrected in parallel.^[^ ^28^ ^]^ Consequently, the reaction, metabolite, and gene information in the model files will need to be corrected accordingly. At this point, the basic SCM consisted of two independent sub‐models with no interactions between them. To address this limitation, we used the model modular assembly function to create new intermediate metabolites and interaction reactions between strains, using external metabolites from the model as templates (Figure 2a). In the model, extracellular metabolites play a crucial role in intercellular interactions since the interactions between strains often involve directed or undirected transfer of certain metabolites. We introduced interaction reaction constraints based on the practicalities of strain culture and exogenous pathways. We also set new external metabolite exchange reactions that are shared by all sub‐models, which ensure the basic uptake of substrates and the output of products.

Take the biosynthesis of sakuranetin by a two‐strain synthetic consortium as an example,^[^ ^9^ ^]^ the metabolic network model of this consortium contained 6154 reactions and 4107 metabolites, compared to the original iML1515 model (2712 reactions and 1877 metabolites).^[^ ^29^ ^]^ As the model size increases exponentially, a larger model can contain more complex information about the reactions. Our models ensure that each module of the system contains the complete metabolic reaction independently, while the interactions are included in the whole synthetic consortia. This approach has the advantage of being both highly generalizable and scalable. Moreover, the labeling of the different sub‐models in the model makes it easier to observe and analyze the flux distribution of the entire system.

#### 2.1.2 Integrating Multiple Constraints for the SCM

The traditional multi‐species metabolic network models just connected and combined different sub‐models with isolated modules for reactions and metabolites. These models only considered stoichiometric constraints and reversibility constraints of reactions, and thus unable to accurately simulate the real metabolic flux distribution of strains. In order to improve the accuracy of simulating the growth states of different strains in a co‐culture system without modifying the complete model information of the sub‐models of different strains, we incorporated independent enzymatic constraints for each sub‐models.

**Figure 3:**
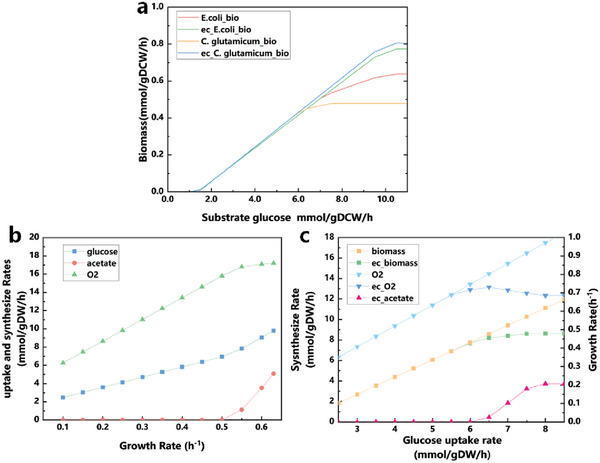
a) The maximum growth rate predicted by different sub‐models in synthetic consortia model. b) Overflow metabolism simulation by *E. coli* sub‐model in co‐cultivated enzyme constraint model. c) Overflow metabolism simulation by *C. glutamicum* sub‐model in co‐cultivated enzyme constraint model.

We constructed a co‐cultivated enzyme constrain model of *E. coli–C. glutamicum* based on the CulECpy method and analyzed the impact of enzyme constraints on the model's prediction of maximum growth rates for different strains. We compared the predicted maximum growth rates of sub‐models in the basic metabolic model and the enzyme‐constrained model, it is evident that enzymatic constraints had a dramatic impact on the predicted growth rates of both strains. When setting glucose as the sole substrate, the maximum growth rates predicted by the enzyme‐constrained model (*E.coli*: 0.64 h^−1^, *C. glutamicum*:0.47 h^−1^) were closer to the wet experimental results (*E.coli*: 0.66 h^−1^, *C. glutamicum*:0.45 h^−1^).^[^ ^30^, ^31^ ^]^ Due to the assumption of a linear relationship between growth rate and substrate uptake rate in the basic metabolic model, accurately simulating the growth of synthetic consortia becomes difficult. However, the co‐cultivated enzyme constrain model sets different enzyme resources for each strain and calculates the enzyme cost (refer to Experimental Section, Equation 4) of crucial reactions to enable independent enzyme constraints for different sub‐models. This allows for a more accurate prediction of growth rates, which is much closer to the wet experimental results (**Figure** **3**a).

In addition, using the co‐cultivated enzyme constraint model, we independently simulated a phenomenon in each strain known as overflow metabolism, where carbon sources are wasted due to the production of byproducts during growth‐up. Overflow metabolism influences the strain's activation of less efficient but low enzyme‐cost fermentation pathways to sustain growth. These pathways consume substrates and generate byproducts such as acetate. This result aligns with previous studies that individually cultured the strain and simulated it using a single‐species model. Overflow metabolism drives the strain to naturally activate fermentation pathways characterized by lower enzyme cost to support growth, leading to the consumption of substrates and the production of byproducts such as acetate (Figure 3b,c). The simulation results are consistent with both the wet experimental results obtained from separate cultivation of the strains and simulations by using single‐species models^[^ ^32^ ^]^.

The CulECpy method, based on the “independent + global” design concept, enables the rapid construction of SCM, which differs from existing modeling methods (**Table** **1**)^[^ ^24^, ^33^, ^34^ ^]^. Our method is applicable to more complex co‐culture metabolic engineering and has been validated using data from several published literatures. We continue to optimize our code to shorten simulation running times as the model scales up. Additionally, we have packaged a python function package to assist users in rapidly modeling different complex SCMs.

**Table 1:** Comparing this method with published methods for microbial community modeling strategies.

Modeling Methods	SEM, SMM, SIM	OptCom	µBialSim	CulECpy
Objective Function	Multi‐scale	Multi‐scale	Multi‐scale	Multi‐scale
Applications	Identification of naturally occurring metabolic relationships; SEM (search for exchanged metabolites), SMM (search for minimal media) , SIM (search for interaction inducing media)	Exploring the interactions within microbial communities	Application to measured data, provide quantitative predictions regarding microbiome dynamics and activity in response to interventions such as changes in the substrate or bioaugmentation	Rational construction of an assisted synthetic consortia, Rational allocation of complex metabolic pathways in synthetic consortia, predicting the optimal initial inoculation ratio for the synthetic consortia, construction of a two‐strain consortium with high yield of 2‐Methylbutyric acid and other bio chemicals
Adaptation	Pairs of genetically engineered mutualistic strains; Dual bacteria system	Capable of accommodating any number of microbial species (or guilds) involved	Natural microbial communities	Synthetic consortia capable of adapting to different strains, any number of microbial species (this paper has verified two‐ and three‐strain co‐culture systems), and complex synthetic pathways for different products
Analysis	Species growth, metabolites shared, induced bacterial relationship, co‐culture minimal media	Species growth, metabolites shared, metabolites consumption/production	Metabolites consumption/production, metabolic Dynamics over time, metabolites shared	Species growth, metabolites shared, metabolites consumption/production, predicting the optimal strain inoculation ratio, optimal route allocation strategy

### 2.2 Function I: Rational Allocation of Complex Metabolic Pathways in Synthetic Consortia

When designing synthetic consortia, researchers need to investigate how the target synthetic pathway is allocated among different strains.^[^ ^35^ ^]^ Common methods are wet experiments for validation, which is guided by empirical methods such as proven pathway strategies in the literature or characterization of intermediate metabolites.^[^ ^36^ ^]^ The whole process requires a significant amount of reagents and labor costs. Therefore, a significant application of SCM lies in employing model simulations to assess various pathway allocation strategies, ultimately reducing experimental costs.

#### 2.2.1 Rational Allocation of Sakuranetin Co‐Culture Synthesis Pathway

**Figure 4:**
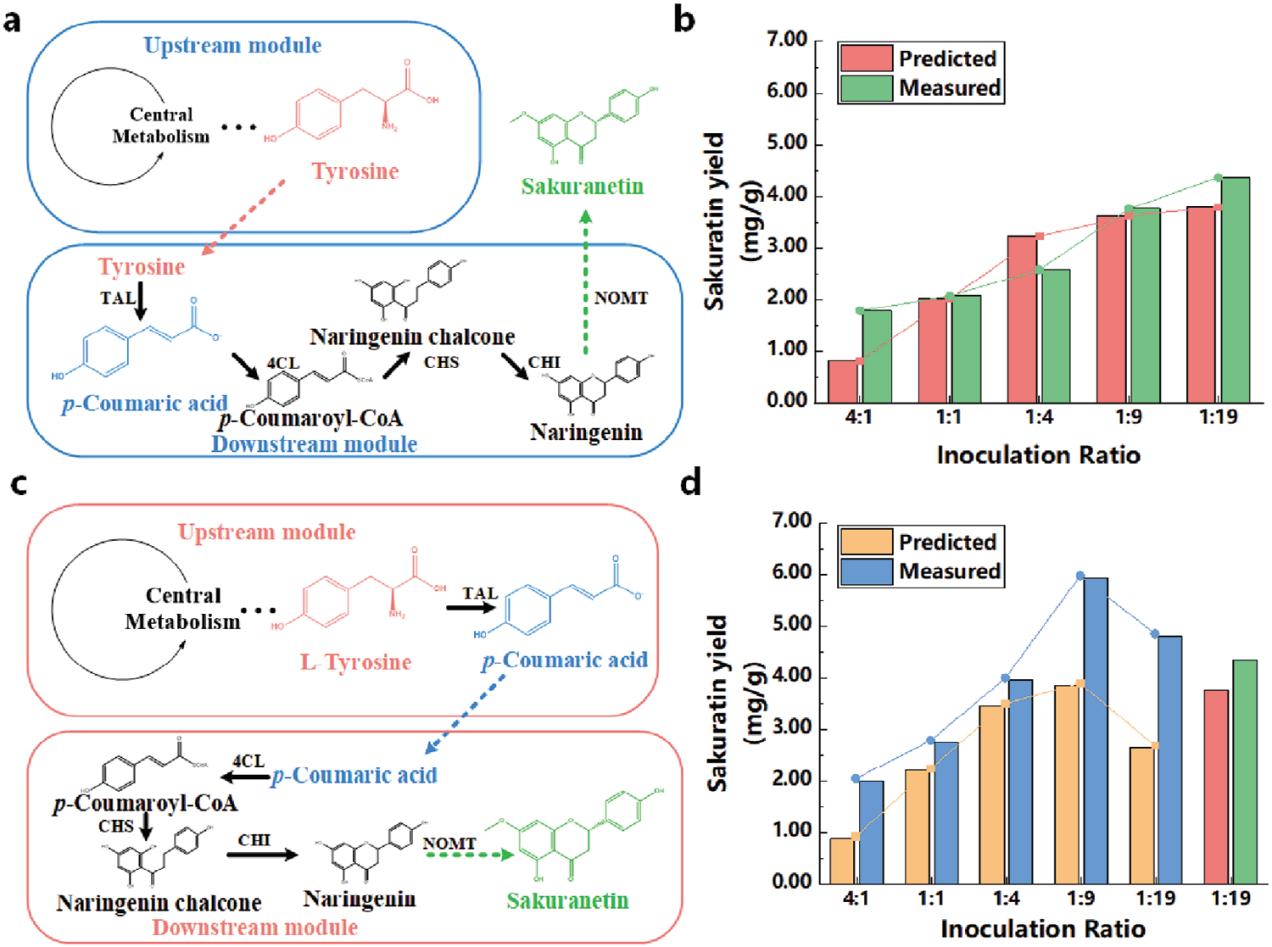
Construction of synthetic consortia for sakuranetin with different pathway allocation strategies. (“predicted” refers to data predicted by the model and “measured” refers to wet experimental data from the literature.) a) Assignment of the sakuranetin synthetic pathway with tyrosine as an intermediate metabolite. b) Two‐strain model simulates the variation of sakuranetin yield with different initial inoculation ratios (Strategy I: tyrosine). c) Assignment of sakuranetin synthetic pathway with *p*‐coumaric acid as an intermediate metabolite. d) Two‐strain model simulates the variation of sakuranetin yield with different initial inoculation ratios (Strategy II: *p*‐coumaric acid).(*TAL* tyrosine ammonia lyase, *4CL* 4‐coumarate: CoA ligase, *CHS* chalcone synthase, *CHI* chalcone isomerase, *NOMT* naringenin 7‐O‐methyltransferase)

Traditional models of synthetic consortia have primarily concentrated on analyzing the complex relationships among strains in the system and macro‐level optimization. However, until recently, there were few methods to specifically analyze differences in co‐culture metabolic pathways. In this work, we constructed a SCM for the synthesis of sakuranetin by synthetic consortia (*E.coli‐E.coli*) using the CulECpy method, and simulated the variation of yield of sakuranetin under different allocation strategies.^[^ ^9^ ^]^ Two different synthetic consortia allocation strategies for the sakuranetin synthesis pathway are shown as **Figure** **4**a,c. In Strategy I (Figure 4a), all the heterologous enzymes, including TAL (tyrosine ammonia lyase), are expressed in the downstream strain, while only the essential tyrosine pathway is left in the upstream module. In Strategy II (Figure 4c), the tyrosine pathway is extended in the upstream module and TAL is used to synthesize *p*‐coumaric acid. Subsequently, *p*‐coumaric acid is transferred as an intermediate metabolite to the downstream module, where sakuranetin is synthesized through a series of exogenous reactions. We used two different pathway assignment strategies to construct separate co‐cultivated enzymatic constraint models and compared them.

We constructed two SCMs using different allocation strategies for the synthesis of sakuranetin. Model I was constructed based on Strategy I, and the simulation results showed that the flux of sakuranetin synthesis continued to increase as the percentage of the downstream module rose from 20% to 95%. The analysis of pathway and flux distribution output revealed that introducing all the heterologous enzymes into the downstream module only increased the metabolic burden of the downstream module. The upstream module did not introduce exogenous reactions and can synthesize sufficient intermediate product tyrosine for the downstream module. In the downstream module, enzyme constraints exerted a greater influence on the reactions, leading to a decrease in the overall flux of the reaction. Consequently, the simulation results demonstrated that a larger proportion of the downstream module was capable of synthesizing sakuranetin, which was consistent with the experimental results.

Model II was constructed based on Strategy II, incorporating exogenous reactions in both the upstream and downstream modules. The simulation results revealed that the sakuranetin yield increased as the proportion of downstream modules increased, reaching a peak at a 1/9 ratio of upstream and downstream modules. Analysis of the simulation flux output revealed a higher percentage of the downstream portion exhibits greater synthesis efficiency, which serves as a critical factor influencing the synthesis of sakuranetin. When the percentage of the upstream module is extremely low, the fluxes of the pathway reach an upper limit and do not synthesize sufficient *p*‐coumaric acid for the downstream module to synthesize sakuranetin. When comparing the maximum theoretical yield of sakuranetin between the two strategies, it has been demonstrated that Strategy II is more suitable as a pathway allocation strategy for the synthesis of sakuranetin in the two‐strain system. The simulation results were consistent with the experimental results of the literature and the trend of the product yield results was highly consistent. The CulECpy method can practically assist in the construction of synthetic consortia and be used to obtain the best pathway allocation strategy as a rational qualitative analysis way.

#### 2.2.2 Rational Allocation of Curcumin Co‐Culture Synthesis Pathway

**Figure 5:**
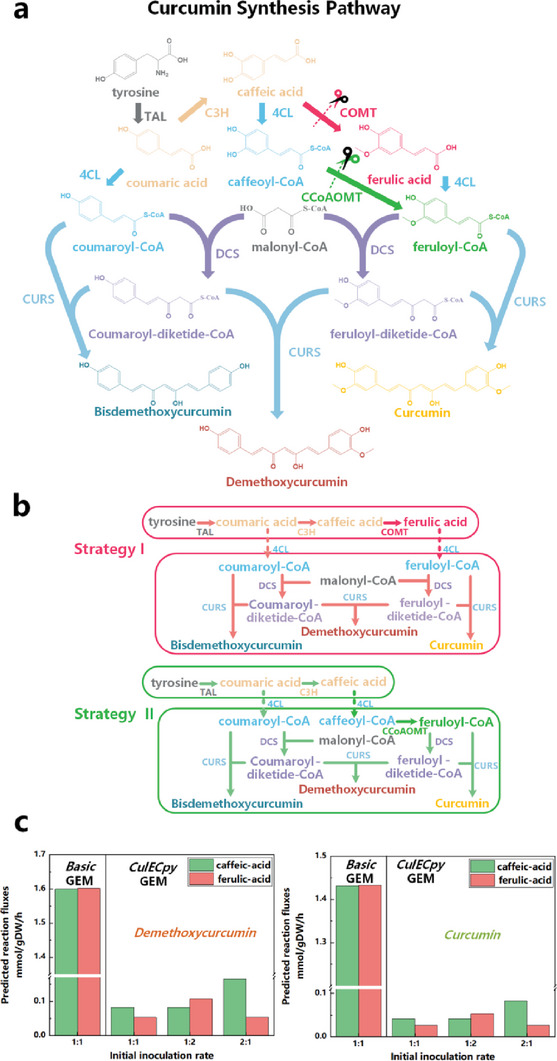
Construction of synthetic consortia for curcumin with different pathway allocation strategies. a) Curcumin synthesis pathway. b) Different strategies to achieve synthetic pathway allocation. c) Simulation of the synthesis capacity of target products (demethoxycurcumin, curcumin) in co‐culture system under different allocation strategies using stoichiometric (Basic GEM) and enzyme‐constrained models (CulECpy GEM).

This study also simulated and analyzed a complex pathway for synthesizing curcumin in a two‐strain co‐culture system (*E.coli–E.coli*).^[^ ^37^ ^]^ The pathway comprises six distinct reactive enzymes, some of which also serve as catalysts for multiple reactions (**Figure** **5**a). Therefore, this method can be employed to simulate and evaluate various pathway allocation strategies to optimize curcumin co‐culture synthesis. We selected the *E. coli* metabolic network model as the foundational framework for our SCM, implementing SCMs construction through two different allocation strategies based on the articles. Strategy I involves the incorporation of the COMT (caffeic acid O‐menthyltransferase) reaction within the upstream module, with caffeic acid serving as the pivotal intermediate metabolite (Figure 5b). On the other hand, Strategy II entails the integration of the CCoAOMT (caffeoyl‐CoA O‐methyl transferase) reaction within the downstream module, utilizing ferulic‐acid as the central intermediate metabolite (Figure 5b). The foundational polymicrobial stoichiometric model and the enzyme‐constrained model derived from the CulECpy method were constructed separately, and employed to evaluate how various pathway assignment strategies impact the capacity for curcumin synthesis.

The results showed that the basic stoichiometric model did not explicitly represent the variations in co‐culture synthesis capacity for different target products (bisdemethoxycurcumin, demethoxycurcumin, curcumin) under different strategies. However, the simulations using the enzyme‐constrained model revealed elevated synthesis fluxes for both demethoxycurcumin and curcumin target products when employing caffeic acid as the intermediate metabolite within the two‐strain co‐culture system. We also conducted a comparative analysis of the effects of the two strategies on reaction fluxes across different modular ratio settings (1:1, 2:1, 1:2; Figure 5c). The comparative findings unveiled that employing Strategy I alongside a 2:1 ratio of upstream to downstream modularization yielded the most optimal curcumin synthesis capacity within the two‐strain co‐culture system. The alignment between our predicted optimal allocation strategy and the experimental observation in the article reaffirms the capacity of our method to qualitatively steer the allocation of intricate synthetic pathways within co‐culture systems, thereby augmenting product synthesis.

#### 2.2.3 Rational Allocation of Non‐Linear Co‐Culture Metabolic Pathways

While linear co‐culture metabolic engineering is more commonly employed in synthetic biology research, non‐linear co‐culture metabolic engineering offers more possibilities for biosynthesis. Researchers need to achieve metabolic equilibrium of all pathway modules in the system, which makes the construction of synthetic consortia more difficult. The CulECpy method is highly scalable and not restricted to essential two‐strain linear pathways. To validate the effectiveness of this method for more complex systems, we carried out simulations using a reference three‐strain non‐linear pathway biosynthesis system for the production of rosmarinic acid (RA).^[^ ^10^ ^]^

**Figure 6:**
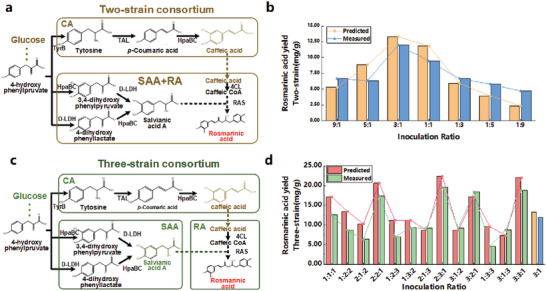
Construction of synthetic consortium of rosmarinic acid with different two/three‐strain pathway design ideas. (“predicted” refers to data predicted by the model and “measured” refers to wet experimental data from the literature.) a) Biosynthesis of the modular metabolic pathway of rosmarinic acid by two‐strain co‐cultures. b) Two‐strain linear co‐culture model simulates rosmarinic acid yield with different initial inoculation ratios. c) Biosynthesis of the modular metabolic pathway of rosmarinic acid by three‐strain co‐cultures. d) Three‐strain nonlinear co‐culture model simulates rosmarinic acid yield with different initial inoculation ratios (and compare the highest rate of two‐strain co‐cultures).

We first constructed an enzyme‐constrained model of the metabolic network for the two‐strain linear pathway involved in the biosynthesis of RA. The upstream module works for the synthesis of the precursor caffeic acid (CA), while the downstream module is constructed by combining the synthesis of salvianic acid A (SAA) and RA (**Figure** **6**a). Next, we adjusted the inoculation ratio of the two modules in the model to establish a metabolic balance in the pathway, aiming to enhance the efficiency of RA synthesis. Through our investigations, we discovered that the optimal ratio occurred when it was set at 3:1. At this ratio, the simulations exhibited a normalized root mean square error of approximately 0.21 compared to the experimental results (Figure 6b). Subsequently, we conducted simulation predictions to explore different reorganizations of the RA, CA, and SAA modules. Interestingly, we found that the two‐strain design combining (CA + RA) and SAA led to an increased metabolic burden, resulting in lower synthesis of caffeic acid and overall lower global efficiency compared to the CA:(SAA + RA) design. The highest theoretical yield obtained 13.27 mg g^−1^ (set the upper bound of substrate glucose uptake rate to 10 mmol g^−1^ DW h^−1^) from the two‐strain linear pathway design model, with an estimated error of 0.096 in comparing the experimental product yields, and the predicted yields for different inoculation ratios were in general agreement with the result of literature.

In this study, we have established a three‐strain SCM utilizing CulECpy (*E.coli–E.coli–E.coli*), based on the pathway design described in the literature, to further explore the potential of modular synthesis of RA. First, we constructed single species GEMs for each of the three modules (CA, SAA, RA) as sub‐models basis. And, these sub‐models were interconnected through a non‐linear pathway to establish interaction reactions (Figure 6c). CulECpy could enhance model simulation and explore the impact of initial module ratios on the synthesis of target products, by integrating partial enzyme constraints and modular scale constraints for each sub‐models. Thirteen initial inoculum ratios of synthetic consortia combinations were selected for simulation analysis, as a more flexible module ratio control strategy is required for modular synthesis in three‐strain systems.

The simulation results for the three‐strain non‐linear system showed a fluctuating pattern of variation, unlike the single trend of the two‐strain system. Specifically, when the inoculum ratio of any one strain was increased, it would affect the flux of the module and the variation of the synthesis of the target product (Figure 6d). Upon further analysis of the flux simulation results obtained from the three‐strain SCM with different ratios, it was observed that the production of RA (the target product) is lower when the ratio of module allocation for CA and SAA is kept at a low level. This observation leads to the conjecture that the limited production of RA is primarily influenced by the bottleneck within the system, which is likely caused by the modules associated with CA and SAA. We tried to conduct an additional simulation experiment in which we adjusted the exogenous fluxes of CA and SAA in the SCM. As a result, the synthetic fluxes of RA were significantly elevated, confirming the above speculation. With the help of CulECpy, the flux distribution and enzyme costs of different reactions in the model were analyzed. It was observed that the CA and SAA modules were indeed more constrained by enzyme kinetics than the RA module. Therefore, the ratio of the RA module is relatively low.

We included a comparison of the RA synthesis flux under a three‐strain co‐cultivation system with another two‐strain allocation strategy ((CA+SAA): RA). Upon comparing these three different strategies, we observed that the three‐strain strategy achieved the optimal yield of RA products. Additionally, we conducted a detailed analysis of the enzyme cost associated with exogenous reactions in various strategies. Each of the three modules had at least one critical reaction (CA: TAL, SAA: D_LDH, RA: 4CL). Therefore, in dual‐strain systems, at least one reaction would be constrained. The three‐strain allocation strategy proved to be the optimal approach for allocating critical reactions in each module. The optimal flux equilibrium for adjusting the ratio between modules was determined by repeatedly simulating the model. The results indicated that the optimal state of CA:SAA:RA was 2:3:1. This specific ratio enabled the SCM to achieve the highest theoretical yield of RA production from glucose consumption, and was identified as the optimal inoculation ratio in the literature. The theoretical yield of the entire set of model simulations was compared with the experimental results in subsequent analyses The normalized root mean square error was less than or equal to 0.25, indicating that the model accurately simulated the undulating results of the target product yield with changes in the initial inoculation ratio.

### 2.3 Function II: Predicting the Optimal Initial Inoculation Ratio for the Synthetic Consortia

Construction of synthetic consortia is a complex research challenge that requires enhanced control over strain interactions. The initial inoculum ratio of strains has a significant impact on co‐culture metabolism, especially as synthetic consortia are dynamic. Consequently, the initial inoculum status is crucial for developing culture optimization strategies. However, traditional metabolic networks cannot accurately predict the optimal inoculation ratio in the synthesis of modular co‐culture to maximize the yields of target products. To address this issue, we developed the CulECpy method with modular interaction scale constraints, which can analyze the differences in the synthetic flux of target metabolites at different initial inoculation rates. It can also predict the inoculation rate at which the theoretical maximum production yield of a given model can be obtained. We constructed co‐cultivated metabolic network models with different strains, substrates, products, or pathway characteristics, and compared them with wet experiments in the literature to globally analyze the trends in target product yield due to different inoculum ratios.

#### 2.3.1 Prediction of the Ratios for Same‐Species Complex Crossover Pathways

**Figure 7:**
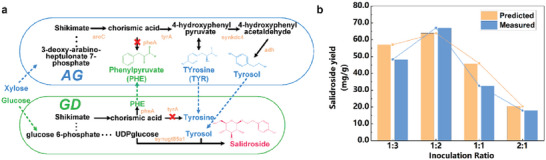
Construction of synthetic consortia for salidroside. (“Predicted” refers to data predicted by the model and “Measured” refers to wet experimental data from the literature.) a) Two‐strain synthesis pathway of salidroside in a complex interleaved. b) Two‐strain model simulates the variation of salidroside yield with different initial inoculation ratios.

In this paper, we validated the application of the SCM construct to a partially complex interleaved co‐culture system. The system involved multiple metabolite interactions between strain module AG and GD, as described in the literature.^[^ ^8^ ^]^ The two groups of strains in the co‐culture system used xylose (AG module) and glucose (GD module) as the main carbon source, respectively, and they constructed a synthetic consortium by cross‐feeding tyrosine and phenylalanine (*E.coli–E.coli*) (**Figure** **7**a).

The model was more difficult to construct than the previous system. First, multiple reactions need to be switched off in the sub‐model to simulate the knockout operation of the specified gene in the literature. Unlike the linear pathway, three new sets of pathway intermediate metabolite interactions need to be created. The optimal ratio of *glucose:xylose* for metabolic equilibrium substrate uptake for salidroside co‐culture synthesis is 4:1, so the upper limit of substrate uptake (EX_glc__D_e_reverse, EX_xyl__D_e_reverse) reaction flux in the model was set at a constraint ratio of 4:1. The model accurately predicted the same trend in the theoretical yield of salidroside as the experimental results. The maximum flux was achieved at an AG:GD ratio of approximately 2:1, with the estimated error of the simulation results being less than or equal to 0.005, and the root mean square error of the product yield normalization for all simulated ratios was 0.19 (Figure 7b). Consequently, the model successfully predicted qualitatively a high convergent production of salidroside optimization strategy with a high fit to the experimental results in the literature.

#### 2.3.2 Prediction of the Ratios for Different‐Species (*E. coli*–*C. glutamicum*) Linear Pathways

**Figure 8:**
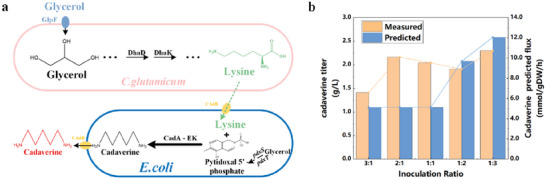
Construction of synthetic consortia for cadaverine. (“predicted” refers to data predicted by the model and “measured” refers to wet experimental data from the literature.) a) *E. coli* and *C. glutamicum* consortium synthesize cadaverine using glycerol as the sole carbon source. b) Two‐strain model simulates the variation of cadaverine yield with different initial inoculation ratios.

To assess the adaptability of CulECpy with various strains of synthetic consortia, we conducted a screening of *E. coli* in combination with *Corynebacterium glutamicum* for cadaverine synthesis(**Figure** **8**a).^[^ ^12^ ^]^ Compared to constructing an SCM with the same strain, the construction of SCM with different strains requires more complex sub‐model modification and parameter optimization workflow. The original GEMs of *E. coli* ^[^ ^29^ ^]^ and *C. glutamicum* ^[^ ^38^ ^]^ were modified to simulate the genetic modification experiments of the prerequisite monocultures.

The two original models were modified with glycerol as the sole carbon source, and target exogenous reactions were added. Enzyme kinetic data files for the two different strain sub‐models were constructed separately and optimized according to the overexpression conditions in the literature design. These files were prepared to ensure the accuracy of the enzyme‐constrained model. Consequently, we successfully constructed a SCM containing *E. coli* and *C. glutamicum*.

Through the rapid construction of an enzyme‐constrained mode, we simulated the theoretical synthetic flux of cadaverine for various strain inoculation ratios. We predicted the optimal initial inoculation ratio (Figure 8b), and it consistent with the culture conditions documented in the literature for reaching the highest cadaverine titer.^[^ ^12^ ^]^ Therefore, CulECpy has the advantage of high adaptability and has the potential to be applied to different strains of synthetic consortia.

## 3 Discussion

Synthetic consortia are creating new opportunities for the green manufacturing of biochemicals. Bottleneck reactions often exist in monoculture systems combined with complex synthetic pathways, directly causing a limitation in the flux of the whole pathway.^[^ ^6^, ^7^, ^8^ ^]^ Synthetic consortia can overcome these limitations by dividing the pathway and adjusting strain ratios, which can effectively reduce the metabolic burden on the strain and adapt complex substrate utilization. Nevertheless, currently, there is a lack of modeling approaches that can facilitate the rapid construction of synthetic consortia. Several modeling techniques that have been developed for microbial communities primarily concentrate on the analysis of intricate strain interactions, without setting product synthesis and modification of metabolic pathways as the key objectives.^[^ ^19^, ^20^, ^21^ ^]^ SCMs have the potential to fulfill several functions. We can rationally allocate of complex synthetic pathways for each strain to improve the substrate conversion efficiency of the whole system and adjust the optimal initial inoculation ratio for strains to achieve the metabolic balance of synthetic consortia. In this study, we proposed the CulECpy method, which provide guidance for the rational construction of synthetic consortia to enhance the biosynthesis of various products.

The CulECpy method integrated the idea of “independent + global” to realize the rapid construction of enzyme‐constrained synthetic consortia metabolic network models based on the traditional single‐species GEMs, and developed simulation analysis functions. With the addition of co‐cultivated enzyme constraints and modular interaction scale constraints, CulECpy strengthen the prediction of the synthesis pathway allocation strategy in synthetic consortia.

We screened several groups of synthetic consortia with different target products, substrate structures or modularity distributions for model construction, and validated them with experimental results to verify the feasibility of CulECpy. For the complex pathway rational allocation, the experimental results of synthesis of sakuranetin were applied to validate the method. The method can accurately simulate the trend of sakuranetin yield generated by two sets of different allocation strategies to construct the model with the change of strain inoculation ratio.^[^ ^9^ ^]^ The simulation results determined that selecting *p*‐coumaric acid as an intermediate allowed for the synthesis of more sakuranetin. Simultaneously, we endeavored to contrast our model with the fundamental stoichiometric model within the framework of the curcumin co‐culture synthesis system.^[^ ^37^ ^]^ The stoichiometry‐based model proved inadequate in simulating the variances in synthesis fluxes resulting from different allocation strategies. Conversely, the enzyme‐constrained model, which we developed for the co‐culture system, effectively evaluated both the preferred pathway allocation strategy and the optimal initial inoculum ratio under these strategy conditions. We also improved the method compatibility and validated the analytical capability in a three‐strain non‐linear synthetic consortium for the synthesis of rosmarinic acid.^[^ ^10^ ^]^ CulECpy accurately predicted the optimal initial inoculation ratio CA:SAA:RA for each module to be 2:3:1, which is consistent with literature results.

The modular interaction scale constraint was integrated into the modeling framework, which had been applied to global flux equilibrium analysis for synthetic consortia, enabled strain modules to be scaled independently, and analysis of the impact of different synthetic consortia compositions on product yield. It was able to simulate the trend variation in product yield for different initial inoculation ratios, and the normalized root mean square error of the comparison experiments were within manageable limits. Significantly, the model exhibits a high level of accuracy in predicting the optimal strain inoculation ratio for co‐culture synthesis. Various synthetic consortia, such as salidroside,^[^ ^8^ ^]^ or cadaverine,^[^ ^11^ ^]^ synthesis system, were selected to verify the accuracy of the method. CulECpy successfully predicted the initial inoculation ratios of modular co‐culture systems such as two‐strain linear co‐culture systems (*E.coli* & *E.coli*, *E.coli* & *C. glutamicum)* ,^[^ ^12^ ^]^ two‐strain complex crossover co‐culture systems^[^ ^8^ ^]^ and three‐strain non‐linear co‐culture systems^[^ ^10^ ^]^ to reach the metabolic equilibrium state. The root mean square error of maximum theoretical yield predicted by SCMs was less than or equal to 0.25.

Opportunities and challenges coexist, the CulECpy method has a typical limitation, which is also found in other SCMs. Models construction relies on basic GEMs and complete enzyme kinetic parameters, and the quality of these parameters directly affects model accuracy. Additionally, a large number of real synthetic consortia are needed to validate and optimize the model. With the broad combination of computer technology and biotechnology, machine learning or deep learning methods are valuable for the construction of models, which can obtain richer enzyme kinetic parameters and build more complex metabolic network models of synthetic consortia. The CulECpy approach is based on the mathematical framework and can derive more functions applicable to synthetic consortia. It also can integrate more complex multi‐omics data and constraints, and combine richer mathematical solving methods to further improve model accuracy.

## 4 Experimental Section

### Basic Model File and Enzyme Kinetic Data Collection for *E. coli*

Basic Model File: The iML1515 and iCW773 GEM^[^ ^39^ ^]^ was chosen as the original model for the integration of various constraints and the construction of various genetically modified *E. coli* (1516 genes, 2712 reactions, and 1877 metabolites)^[^ ^29^ ^]^ and *Corynebacterium.glutamicum* (773 genes, 1207 reactions, and 950 metabolites)^[^ ^38^ ^]^ sub‐models.

Parameters Collection: The enzyme kinetic data k_cat_ are required to achieve the enzyme constraints. In this study, the calibration method in ECMpy^[^ ^40^, ^41^ ^]^ was used to obtain a more complete set of k_cat_ data for enzyme kinetics based on databases such as SABIO‐RK^[^ ^42^ ^]^ and BRENDA.^[^ ^43^ ^]^ The other part of the constraint formula, MocularWeight, uses UniPort,^[^ ^44^ ^]^ and Biocyc^[^ ^45^ ^]^ as direct data sources. The enzyme kinetic data of the heterologous reactions was used during the construction of the SCM, which were based on the reaction EC numbers, reaction substrates, and ambiguous reaction conditions in the above‐mentioned database, and some of the heterologous reactions did not have a valid enzyme kinetic data, we weakened or removed enzyme constraints for these reactions.

### Model Construction Tools

In this study, the Cobrapy Toolbox was used to modify the reaction, metabolite, and gene information in the model files.^[^ ^46^ ^]^ The connection between sub‐models was achieved by creating new external interaction reactions. The Concrete Model framework in the python‐based Pyomo modeling package was used to construct the constraint model^[^ ^47^ ^]^ and selected the Gurobi Solver to calculate all mixed integer linear program. To improve the efficiency of this method, the codes were optimized based on multi‐threaded calls in Python, which significantly reduced the parallel running time of the program to shorten the time costs for researchers.

### Various Constraints for Linear Programming Problems

The basic stoichiometric and rate constraints for flux balance analysis (FBA) were first added:

(1) $$\begin{array}{c} S_{cul}\cdot v_{cul}=0 \end{array}$$

(2) $$0\leq v_{cul}\leq v_{u}$$

A new co‐cultivated metabolic network model was constructed by establishing external metabolite interaction reactions connecting all sub‐models. In the above equation, *S_cul_* represents the stoichiometric matrix of the new model, and *v_cul_* represents the fluxes vector of all reactions, since all reversible reactions are disassembled into irreversible reactions, the lower limit of the reaction fluxes is 0. Equation (1) expresses the flux balance constraint for the global reaction network determined by the stoichiometric matrix, which ensures the participation of each reaction in the global steady‐state calculation in a multi‐species model. The objective function was set to maximize product synthesis, and the growth rate of different strains was limited by enzyme constraints. Building upon this foundation, constraints were added to the sub‐models of different modules based on previously reported enzymatic constraints,^[^ ^40^, ^48^ ^]^ as follows:

(3) $$\sum_{i=1}^{n}\;\frac{v_{x\;i}\cdot MW_{x\;i}}{k_{cat,x\;i}}\leq ENZ_{x}$$

where *x* is number/id of strain.

(4) $$enzyme\;cost_{i}=\frac{v_{x\;i}\cdot MW_{x\;i}}{k_{cat,x\;i}}$$

where *ENZ_x_* denotes the total enzyme variable in sub‐model x, *MW*_*x* *i*_ and *kcat*_*x* *i*_ were the molecular weight and turnover number of an enzyme catalyzing reaction *i* in sub‐model *x*. Different strains may possess different total enzyme pool. In this study, the *ENZ* values for *E. col*i and *C. glutamicum* were calculated using methods described in the literature,^[^ ^40^ ^]^ which rely on the total protein fraction and the mass fraction of enzymes.

The modular interaction scale constraint to the metabolic interaction mechanism between models were innovatively integrated, which achieved quantitative simulation of the interaction flux between different inoculation ratios of strains. Various strains were differentiated within the co‐culture system. To emphasize the impact of the strain ratio on the overall model, the strain community was unitization. As an example of an A, B co‐culture system, if the initial inoculum ratio is *m*: *n*, host A accounts for *m* units of the modular interaction scale and B accounts for *n* units of the modular interaction scale. Setting up the interaction reactions according to this modular interaction scale (Equations 5 and 6), *meta_e_*_*A*_,*meta_e_*_*B*_represent the external metabolites of sub−models A and B, *meta_e_* serves as their shared metabolite, which can circulate freely among them. This was akin to constructing a model for each unit within the modular interaction scale and integrating it into the global flux balance analysis. This constraint was based on the newly established interaction reactions and was ultimately directly incorporated into the stoichiometric matrix of the global metabolic network *S_cul_*.

(5) $$1.0\;meta_{eA}↔\;\frac{1.0}{m}\;meta_{e}$$

(6) $$\frac{1.0}{n}\;meta_{e}↔\;1.0\;meta_{eB}$$

The ratio between the two strain modules in the model is hostA/hostB=m/n, and the parameters *m* and *n* in the constrain limited that the flow of fluxes was simulated in a fixed ratio between sub‐models. We formulated and solved a linear programming problem (LP) by incorporating above constraints and the stoichiometric matrix of metabolic networks. Even with the exponential growth of the model's response information, the complexity of the LPs remains manageable and can be easily solved.

### Simulation

The simulated flux results were first converted into product $yield_{product,s}=\frac{v_{product}*MW_{product}}{v_{substrate}*MW_{substrate}}$, and converted the experimental data results into $yield_{product,e}=\frac{C_{product}}{C_{substrate}}\;$. For comparison of the ability of models to simulate different synthetic consortia, the model and experimental results of product yields were used to calculate the estimation error (Equation 7) and NRMSE (Normalized Root Mean Square Error, NRMSE, Equation 8).

(7) $$Estimation\;error\;=\frac{\left|yield_{product,s}-yield_{product,e}\right|}{yield_{product,e}}$$

(8) $$Yield\;NRMSE\;=\frac{\sqrt{\sum_{ratio}{\left(yield_{product,s_{ratio}}-yield_{product,e_{ratio}}\right)}^{2}}}{\left|yield_{product,max}-yield_{product,min}\right|}\;$$

*ratio: Inoculation ratio of different strains*

## Conflict of Interest

The authors declare no conflict of interest

## Author Contributions

B.X. contributed in conceptualization, method design and code implementation, data curation, writing—original draft, Writing—review and editing. Y.L. performed guidance on research related to synthetic consortia. C.Y. contributed in algorithm, methodology. H.L. contributed in data collection. Q.Y. contributed in algorithm and methodology. S.W. contributed in methodology and software. H.S. contributed in conceptualization and writing—review and editing.

## Data Availability

The data that support the findings of this study are available from the corresponding author upon reasonable request.;

## References

[1] B. M. Woolston , S. Edgar , G. Stephanopoulos , Annu. Rev. Chem. Biomol. Eng. 2013, 4, 259. 23540289 10.1146/annurev-chembioeng-061312-103312

[2] A. S. Khalil , J. J. Collins , Nat. Rev. Genet. 2010, 11, 367. 20395970 10.1038/nrg2775 PMC2896386

[3] H. Zhang , X. Wang , Metab. Eng. 2016, 37, 114. 27242132 10.1016/j.ymben.2016.05.007

[4] C. R. Lee , C. Kim , Y. E. Song , H. Im , Y.‐K. Oh , S. Park , J. R. Kim , Bioresour. Technol. 2018, 259, 128. 29549832 10.1016/j.biortech.2018.02.129

[5] Y. Liu , X. Tu , Q. Xu , C. Bai , C. Kong , Q. Liu , J. Yu , Q. Peng , X. Zhou , Y. Zhang , M. Cai , Metab. Eng. 2018, 45, 189. 29258964 10.1016/j.ymben.2017.12.009

[6] R. Wang , S. Zhao , Z. Wang , M. A. Koffas , Curr. Opin. Biotechnol. 2020, 62, 65. 31605875 10.1016/j.copbio.2019.09.004

[7] A. D. Flores , E. Z. Ayla , D. R. Nielsen , X. Wang , ACS Synth. Biol. 2019, 8, 1089. 30979337 10.1021/acssynbio.9b00007

[8] X. Liu , X.‐B. Li , J. Jiang , Z.‐N. Liu , B. Qiao , F.‐F. Li , J.‐S. Cheng , X. Sun , Y.‐J. Yuan , J. Qiao , G.‐R. Zhao , Metab. Eng. 2018, 47, 243. 29596994 10.1016/j.ymben.2018.03.016

[9] X. Wang , Z. Li , L. Policarpio , M. A. G. Koffas , H. Zhang , Appl. Microbiol. Biotechnol. 2020, 104, 4849. 32285175 10.1007/s00253-020-10576-1

[10] Z. Li , X. Wang , H. Zhang , Metab. Eng. 2019, 54, 1. 30844431 10.1016/j.ymben.2019.03.002

[11] R. Kawai , Y. Toya , K. Miyoshi , M. Murakami , T. Niide , T. Horinouchi , T. Maeda , A. Shibai , C. Furusawa , H. Shimizu , Biotechnol. Bioeng. 2022, 119, 936. 34914093 10.1002/bit.28007

[12] S. Liu , J. Mi , K. Song , H. Qi , L. Zhang , Biotechnol. Lett. 2022, 44, 1389. 36203106 10.1007/s10529-022-03306-2

[13] X. Fang , C. J. Lloyd , B. O. Palsson , Nat. Rev. Microbiol. 2020, 18, 731. 32958892 10.1038/s41579-020-00440-4 PMC7981288

[14] C. Ye , Q. Luo , L. Guo , C. Gao , N. Xu , L. Zhang , L. Liu , X. Chen , Biotechnol. Bioeng. 2020, 117, 3533. 32648933 10.1002/bit.27485

[15] R. Agren , J. M. Otero , J. Nielsen , J. Ind. Microbiol. Biotechnol. 2013, 40, 735. 23608777 10.1007/s10295-013-1269-3

[16] P. Xu , Q. Gu , W. Wang , L. Wong , A. G. W. Bower , C. H. Collins , M. A. G. Koffas , Nat. Commun. 2013, 4, 1409. 23361000 10.1038/ncomms2425

[17] W. Jiang , J. B. Qiao , G. J. Bentley , D. Liu , F. Zhang , Biotechnol. Biofuels 2017, 10, 244. 29090017 10.1186/s13068-017-0936-4 PMC5658922

[18] C. V. Dinh , X. Chen , K. L. J. Prather , ACS Synth. Biol. 2020, 9, 590. 32040906 10.1021/acssynbio.9b00451

[19] R. Guillonneau , C. Baraquet , A. Bazire , M. Molmeret , Front. Microbiol. 2018, 9, 1960. 30214432 10.3389/fmicb.2018.01960 PMC6125326

[20] J. Naylor , H. Fellermann , Y. Ding , W. K. Mohammed , J. Mukherjee , C. A. Biggs , P. C. Wright , N. Krasnogor , ACS Synth. Biol. 2018, 7, 952. 29493230 10.1021/acssynbio.8b00077

[21] B. García‐Jiménez , J. Torres‐Bacete , J. Nogales , Comput. Struct. Biotechnol. J. 2021, 19, 226. 33425254 10.1016/j.csbj.2020.12.003 PMC7773532

[22] D. Reiman , B. T. Layden , Y. Dai , PLoS Comput. Biol. 2021, 17, e1009021. 33999922 10.1371/journal.pcbi.1009021 PMC8158931

[23] C. Diener , S. M. Gibbons , O. Resendis‐Antonio , mSystems 2020, 5, e00606. 31964767 10.1128/mSystems.00606-19 PMC6977071

[24] A. R. Zomorrodi , C. D. Maranas , PLoS Comput. Biol. 2012, 8, e1002363. 22319433 10.1371/journal.pcbi.1002363 PMC3271020

[25] S. H. J. Chan , M. N. Simons , C. D. Maranas , PLoS Comput. Biol. 2017, 13, e1005539. 28505184 10.1371/journal.pcbi.1005539 PMC5448816

[26] H. Song , M.‐Z. Ding , X.‐Q. Jia , Q. Ma , Y.‐J. Yuan , Chem. Soc. Rev. 2014, 43, 6954. 25017039 10.1039/c4cs00114a

[27] Q. Ma , Y.‐H. Bi , E.‐X. Wang , B.‐B. Zhai , X.‐T. Dong , B. Qiao , M.‐Z. Ding , Y.‐J. Yuan , J. Ind. Microbiol. Biotechnol. 2019, 46, 21. 30368638 10.1007/s10295-018-2096-3

[28] Y. Liu , N. Xie , B. Yu , ACS Synth. Biol. 2022, 11, 1361. 35244401 10.1021/acssynbio.2c00007

[29] J. M. Monk , C. J. Lloyd , E. Brunk , N. Mih , A. Sastry , Z. King , R. Takeuchi , W. Nomura , Z. Zhang , H. Mori , A. M. Feist , B. O. Palsson , Nat. Biotechnol. 2017, 35, 904. 29020004 10.1038/nbt.3956 PMC6521705

[30] N. Okahashi , S. Kajihata , C. Furusawa , H. Shimizu , Metabolites 2014, 4, 408. 24957033 10.3390/metabo4020408 PMC4101513

[31] Z. Wang , J. Liu , L. Chen , A.‐P. Zeng , C. Solem , P. R. Jensen , Metab. Eng. 2018, 48, 1. 29753071 10.1016/j.ymben.2018.05.004

[32] M. J. Van Hoek , R. M. Merks , BMC Syst. Biol. 2012, 6, 22. 22443685 10.1186/1752-0509-6-22 PMC3384451

[33] D. Popp , F. Centler , Front. Bioeng. Biotechnol. 2020, 8, 574. 32656192 10.3389/fbioe.2020.00574 PMC7325871

[34] N. Klitgord , D. Segrè , PLoS Comput. Biol. 2010, 6, e1001002. 21124952 10.1371/journal.pcbi.1001002 PMC2987903

[35] J. A. Jones , V. R. Vernacchio , S. M. Collins , A. N. Shirke , Y. Xiu , J. A. Englaender , B. F. Cress , C. C. McCutcheon , R. J. Linhardt , R. A. Gross , M. A. G. Koffas , mBio. 2017, 8, e00621. 28588129 10.1128/mBio.00621-17 PMC5461408

[36] K. Zhou , K. Qiao , S. Edgar , G. Stephanopoulos , Nat. Biotechnol. 2015, 33, 377. 25558867 10.1038/nbt.3095 PMC4867547

[37] J. L. Rodrigues , D. Gomes , L. R. Rodrigues , Front. Bioeng. Biotechnol. 2020, 8, 59. 32117938 10.3389/fbioe.2020.00059 PMC7019186

[38] Y. Zhang , J. Cai , X. Shang , B. Wang , S. Liu , X. Chai , T. Tan , Y. Zhang , T. Wen , Biotechnol. Biofuels 2017, 10, 169. 28680478 10.1186/s13068-017-0856-3 PMC5493880

[39] Z. A. King , J. Lu , A. Dräger , P. Miller , S. Federowicz , J. A. Lerman , A. Ebrahim , B. O. Palsson , N. E. Lewis , Nucleic Acids Res. 2016, 44, D515. 26476456 10.1093/nar/gkv1049 PMC4702785

[40] Z. Mao , X. Zhao , X. Yang , P. Zhang , J. Du , Q. Yuan , H. Ma , Biomolecules 2022, 12, 65. 35053213 10.3390/biom12010065 PMC8773657

[41] J. Niu , Z. Mao , Y. Mao , K. Wu , Z. Shi , Q. Yuan , J. Cai , H. Ma , Biomolecules 2022, 12, 1499. 36291707 10.3390/biom12101499 PMC9599660

[42] U. Wittig , M. Rey , A. Weidemann , R. Kania , W. Müller , Nucleic Acids Res. 2018, 46, D656. 29092055 10.1093/nar/gkx1065 PMC5753344

[43] S. Placzek , I. Schomburg , A. Chang , L. Jeske , M. Ulbrich , J. Tillack , D. Schomburg , Nucleic Acids Res. 2017, 45, D380. 27924025 10.1093/nar/gkw952 PMC5210646

[44] The UniProt Consortium , Nucleic Acids Res. 2015, 43, D204. 25348405 10.1093/nar/gku989 PMC4384041

[45] P. D. Karp , R. Billington , R. Caspi , C. A. Fulcher , M. Latendresse , A. Kothari , I. M. Keseler , M. Krummenacker , P. E. Midford , Q. Ong , W. K. Ong , S. M. Paley , P. Subhraveti , Brief. Bioinform. 2019, 20, 1085. 29447345 10.1093/bib/bbx085 PMC6781571

[46] A. Ebrahim , J. A. Lerman , B. O. Palsson , D. R. Hyduke , BMC Syst. Biol. 2013, 7, 74. 23927696 10.1186/1752-0509-7-74 PMC3751080

[47] W. E. Hart , J.‐P. Watson , D. L. Woodruff , Math. Program. Comput. 2011, 3, 219.

[48] P. S. Bekiaris , S. Klamt , BMC Bioinformatics 2020, 21, 19. 31937255 10.1186/s12859-019-3329-9 PMC6961255

